# Impact of Processing and Intestinal Conditions on in Vitro Digestion of Chia (*Salvia hispanica*) Seeds and Derivatives

**DOI:** 10.3390/foods9030290

**Published:** 2020-03-05

**Authors:** Joaquim Calvo-Lerma, Carolina Paz-Yépez, Andrea Asensio-Grau, Ana Heredia, Ana Andrés

**Affiliations:** 1 Instituto de Ingeniería de Alimentos para el Desarrollo, Universitat Politècnica de València, 46022 Valencia, Spain; capaye@doctor.upv.es (C.P.-Y.); anasgr@upv.es (A.A.-G.); anhegu@tal.upv.es (A.H.);; 2 Facultad de Ciencias Agrarias, Universidad Agraria del Ecuador, 090150 Guayaquil, Ecuador

**Keywords:** chia, chia seeds, chia flour, sprouting, milling, lipolysis, proteolysis, antioxidant activity, in vitro digestion, pancreatic insufficiency

## Abstract

Chia seeds present with an excellent nutrient profile, including polyunsaturated fat, protein, fibre and bioactive compounds, which make them a potential food or ingredient to bring beneficial health effects. However, their tough structure could mean that these seeds remain hardly disrupted during digestion, thus preventing the release and digestibility of nutrients. In the present study, different chia products (seeds, whole flour, partially defatted flour and sprouts) were assessed in terms of proteolysis, lipolysis, calcium and polyphenols bioaccessibility and antioxidant activity. In vitro digestions were performed supporting standard intestinal (pH 7, bile salts concentration 10 mM) and altered (pH 6, bile salts concentration 1 mM) conditions. The altered conditions significantly reduced lipolysis, but not proteolysis. Regarding the food matrix, compared to the chia seeds, whole and partially defatted flour increased the hydrolysis of lipids and protein, relating to reduced particle size. Sprouting had an enhancing effect on proteolysis but prevented lipolysis. Calcium bioaccessibility dropped in all the samples in the two intestinal conditions. The digestion process led to increased polyphenols bioaccessibility in all the structures, but reduced antioxidant activity except in the milled structures. In conclusion, milling should be applied to chia seeds prior to consumption in cases where enhancing the potential uptake of macro and micronutrients is targeted, and sprouting is suitable to enhance protein digestibility and reduce lipolysis.

## 1. Introduction

Chia (*Salvia hispanica L.*) is an herbaceous plant that has been traditionally consumed by Mesoamerican populations, and is nowadays becoming popular in Europe and North America [1]. The nutrient composition of chia seeds makes it a highly appreciated functional ingredient and a recommended food as part of healthy diets [2]. Chia presents with high amounts of linolenic acid (omega 3), which relates to anti-inflammatory effects. Additionally, chia seeds are rich in dietetic fibre, protein of high biological value, calcium and antioxidant micronutrients [3]. The main phenolic compounds include chlorogenic acid and caffeic acid, which protect against free radicals and inhibit lipid and DNA peroxidation, and myricetin, quercetin and kaempferol, which are related to cardiovascular protecting effects [4]. 

In this sense, the European Commission extended the use of chia as a new food ingredient in bakery, breakfast cereal and fruit, nuts and seeds mixture products [5], which encouraged numerous studies regarding the characterisation and incorporation of chia seeds into different formulations. Inglett, Chen, Liu, and Lee (2014) concluded that chia seeds alone are difficult to integrate into food products because of the small size, hard cover and low cohesiveness [6]. Indeed, the external layer of the seeds may make lipid and protein hydrolysis difficult, and hinder the bioactive compound release of the matrix, further reducing solubilisation with the digestive fluids, limiting bioavailability overall [7]. 

Therefore, alternatives to the direct consumption of chia seeds could be recommended. Some processes such as soaking, sprouting or fermentation could increase its digestibility. From these alternatives, sprouting has gained popularity as it is an economic and simple method that allows for improving the nutritional value of the seeds, including increased phenols and mineral content [8,9]. Similarly, the milling of seeds has also been proven to enhance nutrient digestibility because of matrix disruption and ease of nutrient release from the interior of plant cells [10]. However, little scientific evidence can support a beneficial effect of different treatments of chia seed regarding lipolysis, proteolysis and the bioavailability of micronutrients such as calcium or phytochemicals such as phenols. 

In addition to the food matrix structure, intestinal conditions could affect nutrient digestibility [11]; some pathologies, such as exocrine pancreatic insufficiency, cystic fibrosis and hepatic alterations are related to decreased secretion of digestive fluids, overall leading to suboptimal intestinal conditions [12,13,14,15]. Previous studies have shown that particularly low duodenal pH and reduced bile salts concentration are determinants in the fate of lipid and protein digestion [11,16,17,18,19,20].

In this context, the aim of the present study was the characterization of lipid and protein digestibility, along with the assessment of phenolic compounds and calcium bioaccessibility in different chia seed products by means of an in vitro digestion methodology. Secondarily, assessing the effect of altered intestinal conditions was also targeted.

## 2. Materials and Methods 

### 2.1. Reagents

The simulated digestive fluids to conduct in vitro digestions were prepared with KCl, KH_2_PO_4_, NaCl, NaHCO_3_, MgCl_2_ (H_2_O)_6_, (NH_4_)_2_CO_3_, CaCl_2_, human α-amylase (1000–3000 U/mg protein), pepsin from porcine gastric mucosa ( ≥ 2500 U/mg protein) and bovine bile extract, all of which were obtained from Sigma-Aldrich Chemical Company (St Louis, MO, USA). The pancreatic enzyme supplements came from Kreon 10,000 LU (Mylan, Canonsburg, PA, USA). For the analytical determinations, Triton-X 100%, trichloroacetic acid (TCA), hexane, Folin-Ciocalteu reagent, Na_2_CO_3_, gallic acid (GA), trolox (TX) and the analytical standards were acquired from Sigma-Aldrich Chemical Company (St Louis, MO, USA). Ethanol (95% *v/v* for analysis), NaOH and HCl, were from AppliChem Panreac (Barcelona, Spain).

### 2.2. Chia Products

Chia seeds and three derivatives were assessed in the present study. The seeds were subjected to germination on the one hand, resulting in chia sprouts; and on the other hand, milling was applied to obtain whole chia flour. Additionally, commercial partially defatted chia seed flour was studied. The chia seeds and partially defatted chia flour were purchased at a local supermarket (Valencia, Spain). 

The rationale behind this selection was as follows: (a) the interest in evaluating the sprouting effect in nutrient composition and digestibility; (b) analysing the influence of particle size, comparing the behaviour against in vitro digestion between whole seeds and flour and (c) evaluating the impact of partially defatted chia flour on the digestibility and bioaccessibility of nutrients. 

#### 2.2.1. Milling Process for Chia Flour

Whole chia flour was obtained with a mechanic grinder (Taurus Aromatic SP-7407 50 Hz, Ø80 mm grinding dial at 1480 rpm; Lleida, Spain) by applying a constant pulse for 15 s until the particle was equivalent to partially defatted commercial chia seed flour.

Partially defatted chia flour was purchased as such; this product is known to be obtained as a by-product in the industrial production of chia oil. After the mechanical pressing of chia seeds and extracted oil removal, the remaining parts are rinsed with water and dried, this resulting product being the commercial partially defatted chia flour [21].

#### 2.2.2. Seeds Germination for Chia Sprouts

To obtain chia sprouts, an electric seed germinator was used (Easygreen EGL55, Seed and Grain Technologies, Inc., Danbury, CT, USA), which includes a time-controlled nebulizer that enables water spraying and oxygenation settings according to the desired conditions for specific seeds sprouting. The germination process was carried out according to [22] with some amendments. Chia seeds were arranged on sterile trays and water sprayed with deionized water twice a day at 22 ± 2 °C. After 10 days of growing, sprouted seeds were harvested.

### 2.3. In Vitro Digestion

Chia seeds and derivatives were subjected to in vitro digestion. Experiments were conducted following the static in vitro digestion model that allows for simulating both standard and altered intestinal conditions [16] concretely in terms of duodenal pH and bile salts concentration [11].

The method consisted of the simulation of the oral, gastric and small intestinal stages by means of the application of mechanical forces, digestive fluids and temperature. Table 1 gathers the summary of the conditions used at the different in vitro digestion stages. Chia seeds were digested as intact structures on the one hand, and on the other hand chia seeds and sprouts were digested as partially disintegrated samples after simulating mastication according to Mandalari et al. [23]. As for the other chia derivatives, the two flours (whole chia flour and partially defatted chia flour) skipped the simulated mastication, as both were already completely unstructured. The simulation of the altered intestinal conditions was conducted at intestinal pH 6 and a bile salts concentration of 1 mM [11,13,14,15,24], while the standard digestion of a healthy individual was simulated with intestinal pH 7 and a bile salts concentration of 10 mM [25,26]. At the end of the intestinal stage, the undigested fraction was separated by centrifugation (4000× *g* for 15 min) and aliquots from the supernatant (bioaccessible fraction) were used for analytical determinations. All the experiments were conducted in triplicate.

After the in vitro digestion process, the digestibility of lipid and protein was defined as the extent of lipid and protein hydrolysis respectively. The bioaccessibility of micronutrients and phytochemicals was used to express the fraction of the total amount of calcium and polyphenols that was released from the matrix and, therefore, potentially available for absorption.

### 2.4. Analytical Determinations

#### 2.4.1. Moisture, Lipid, Protein and Carbohydrates

AOAC methodologies were followed to determine the chemical composition of the chia seeds and sprouts (AOAC 2000) [27]: lipid content was quantified by the Soxhlet extraction method, protein by Kjeldhal digestion and moisture by sample drying and gravimetry; carbohydrates were calculated considering total solids and subtracting lipid and protein.

#### 2.4.2. Proteolysis

Proteolysis was determined by assessing the tricloracetic acid-soluble protein fraction (TCA) at the end of the gastric and the intestinal stage following the protocol described by Lamothe, Azimy, and Bazinet (2014) [28], and absorbance (optical density, OD) at 280 nm was measured, using a spectrophotometer (UV/vis, Beckman Coulter), against a blank prepared with the appropriate digestive fluids [29].

#### 2.4.3. Lipolysis

Free fatty acids (FFA) released after intestinal digestion were measured to quantify lipolysis extent by means of a spectrophotometric assay kit (Roche Diagnostics, Indianapolis, IN, USA) in a UV/vis spectrophotometer (Beckman Coulter), as previously described by Lamothe et al. [30], measuring absorbance at 546 nm. The result was expressed as lipolysis extent (%) which was estimated assuming the release of 2 moles of fatty acids per 1 mol of triglyceride, and considering the molecular weight of the majoritarian fatty acid in chia [28].

#### 2.4.4. Calcium

The calcium content in the initial and digested samples was determined by flame atomic absorption spectrometry (Thermo Scientific, iCE 3000 Series, Pittsburgh, PA, USA). Samples of the initial (6 g) and digested products (1 mL of bioaccessible fraction) were incinerated. The obtained ashes were dissolved in 1.5 mL of extra pure nitric acid (65%) and 4 mL of distilled water were added. Then, 2.5 mL of lanthan (1%) were incorporated to prevent reading interferences in the equipment. Finally, distilled water was added to reach a total solution volume of 25 mL. Absorbance was measured at 422.7 nm adjusting zero with a blank sample (La^3+^ solution 0.1%) [31,32].

#### 2.4.5. Polyphenols

Total polyphenols were determined according to the modified protocol of Paz-Yepez et al. [19,33]. Polyphenols extraction from the lyophilised sample (50 mg) was conducted with 1 mL of methanol (70:30 methanol-water (*v*/*v*)) in agitation at 55 rpm during 120 min at 25 °C (intel-Mixer RM-2, Emi Ltd., Riga, LV-1006, Letonia). The mixture was thereafter centrifugated at 14.1× *g* for 20 min (Eppendorf Minispin^®^, Eppendorf Ibérica S.L.U., Madrid, Spain). Then, the methanolic extract (125 µL) was added to a 4 mL plastic cuvette with distilled water (0.5 mL) and the Folin-Ciocalteu reagent (125 µL). After 5 min, 1.25 mL of Na_2_CO_3_ (7% (*w*/*v*)) and distilled water (1 mL) were added, and absorbance was measured at 760 nm. The calibration curve was made with gallic acid (0–700 µmol gallic acid (GA)/L) as standard. The results were expressed as mg of equivalent gallic acid per gram of food and dry matter.

#### 2.4.6. Antioxidant Activity

Spectrophotometric determination of the 2,2-diphenil-1-pricrilhidayil (DPPH) radical was used to measure total antioxidant activity. This method is founded on the capacity an antioxidant compound has to neutralise the DPPH radical [34]. The DPPH solution was prepared by dissolving 35 mg DPPH reagent with 1000 mL of methanol to obtain an absorbance of 1.1 ± 0.02. The extraction was carried out as described for polyphenols, applying a centrifugation at 4000× *g* for 20 min. Following this, 30 µL of methanolic extracts were allowed to react for 60 min with 3 mL of DPPH solution. Then, the absorbance was measured at 515 nm. 

#### 2.4.7. Particle Size Measurement

After mechanical disruption in the oral stage, samples were measured for particle size distribution using a laser light scattering instrument (Mastersizer 2000, Malvern Instruments Ltd., Malvern, UK). The instrument measured the angular dependence of the intensity of laser light diffraction and assigned the particle size distribution that gives the best fit to the experimental measurements and predictions, based on light scattering theory. The mean particle size was reported as the surface weighted mean diameter, d_3,2_ [35].

### 2.5. Statistical Anlysis 

Data were summarised as mean and standard deviation. Simple ANOVA analyses were performed to assess the statistical significance of the processes applied to chia (milling, defatting and sprouting) on nutritional composition and particle size; and these processes and the intestinal conditions on proteolysis, lipolysis, calcium and polyphenols bioaccessibility and antioxidant activity. Statgraphics Centurion was used, and the analyses were conducted with at least a significance of 95% (*p*-value < 0.05).

## 3. Results

### 3.1. Chia Structures Matrix Characterisation

Before subjecting the matrices to in vitro digestion, the four different chia products were characterised for nutrient composition in terms of moisture, lipids, protein and calcium content. In addition, polyphenol compounds and total antioxidant activity were determined. In Table 2 the results are expressed in dry basis, in order to evidence the impact of the processes applied to chia seeds (milling, defatting and germination) on these compounds. As observed, the main differences relate to chia sprout as compared to the chia seed matrices, concretely as far as moisture is concerned (from around 0.065 in seeds to 9.87 g/g dry matter in the sprouts). When considering the composition in the dry basis, a reduction in lipid content was observed in the chia sprouts, along with an increase in the content of protein, carbohydrate and polyphenols and an increment in antioxidant activity.

**Figure 1:**
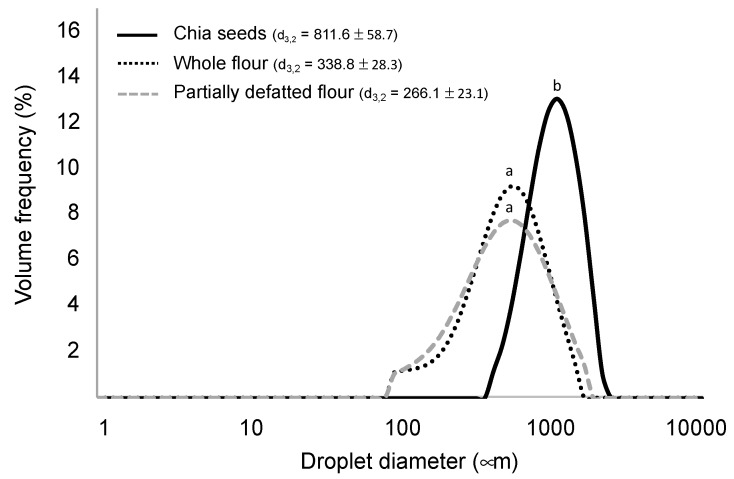
Particle size distributions of the chia seeds and chia flours imparted by the mechanical process and the simulated mastication. d_3,2_ values sharing a same letter are not significantly different (*p* < 0.05) (data are expressed as mean values from three repetitions).

Moreover, in a prior gastrointestinal digestion simulation, the particle sizes of chia seeds and flours were determined in order to define the samples in terms of structural characteristics. As shown in Figure 1, chia seeds, after undertaking the oral stage, resulted in a mean particle diameter of 811.6 µm, which, as expected, was higher than in the completely unstructured flours, with both showing similar particle sizes (d_3,2_ being 338 and 266 µm in the whole flour and in the partially defatted flour, respectively). These results may be useful to understand the nutrient digestibility and bioaccessibility results in the coming sections, as nutrient release from the matrix is highly dependent on the degree of matrix disruption [10,18,36].

**Figure 2:**
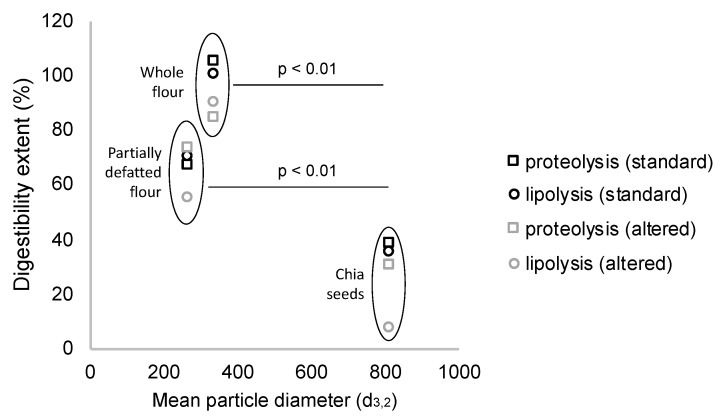
Milling process of chia seeds reduces particle size, which significantly increases lipid and protein digestibility in chia flours as compared to chia seeds (after simulation of mastication) (data are expressed as mean values from three repetitions).

When assessing protein and lipid digestibility, the particle size of the different structures resulted in exerting a significant effect, so that lower particle sizes of the chia structures were associated with higher levels of proteolysis and lipolysis (Figure 2). Accordingly, both whole and partially defatted flours achieved significantly higher lipolysis extents than chia seeds. These matrices were highly disrupted, so the components of the seeds were supposed to be available as enzyme substrates. However, the partially defatted flour, despite having slightly smaller particle size, had lower digestibility extents. The possible reasons for this finding relate to different processing steps involved in the defatting process, which could have affected protein structure [37], and overall, led to the smaller ratio lipid/protein.

### 3.2. Lipid and Protein Digestibility

**Figure 3:**
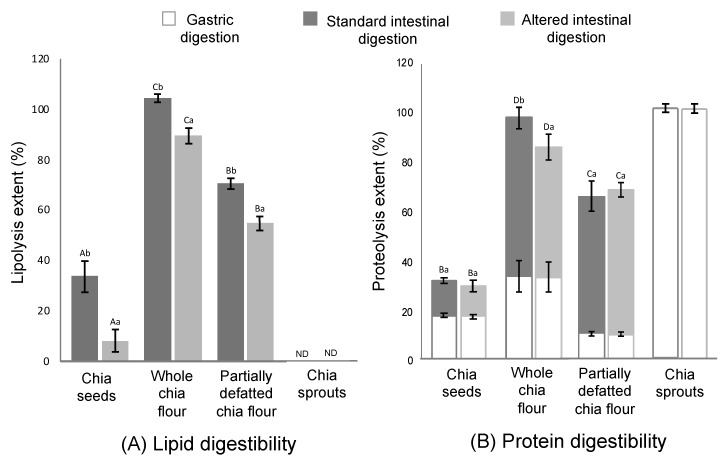
Lipid (**A**) and protein (**B**) digestibility in chia seeds, whole chia flour, partially defatted chia flour and chia sprouts during the gastric and standard (pH 7, bile salts concentration 10 mM) and altered (pH 6, bile salts concentration 1 mM) intestinal conditions. ND = non-detectable. Different capital letters (A–D) mean significant differences (*p* < 0.05) between chia products (chia seeds, whole chia flour, partially defatted chia flour and chia spouts). Different lowercase letters (a, b) means significant differences (*p* < 0.05) between intestinal conditions (standard and alternated) (data are expressed as mean values from three repetitions and the error bars represent the standard deviation).

Whole chia flour reached the maximum lipolysis extents among all the study matrices, namely 100% in the normal and 90% in the altered conditions (Figure 3A). Focusing on proteolysis (Figure 3B), the gastric stage accounted for extents around 10%–35% in chia seed structures, while 100% was achieved in chia sprouts. In the intestinal stage, maximum proteolysis was achieved by whole chia flour, followed by partially defatted flour and chia seeds. In addition to the study of nutrient digestibility in the four study matrices commented on in the present section, the assessment of intact chia seeds (skipping the mastication simulation) was targeted. As expected, both the lipid and protein digestibility in intact chia seeds were null.

### 3.3. Bioaccessibility of Calcium and Polyphenols and Antioxidant Activity

Other relevant components in chia include the micronutrients and phytochemicals, with calcium and polyphenols representing the larger proportions. The results of calcium and polyphenols concentrations, along with the antioxidant activity, after in vitro digestion are gathered in Table 3.

Compared to cow milk, chia seeds are a potentially good source of this micronutrient. In the present study, different amounts of calcium were registered among the studied chia products, but when it comes to calcium concentration in the absorbable fraction after in vitro digestion, all of the samples experimented a significant decrease, with no significant influence of the intestinal conditions The highest drop was observed in digested chia sprouts, with a net variation of about 96%. The total polyphenols and antioxidant activity of the different chia products were assessed after in vitro digestion under normal and altered conditions. The values of polyphenols concentrations in the absorbable fraction of the digested samples reveal a significant change in the extractability of these compounds as consequence of the gastrointestinal digestion. Digestion under standard intestinal conditions allowed for higher polyphenol extractability than the altered conditions. Finally, antioxidant activity demonstrated a decrease after digestion in all the cases, being sharper in altered conditions than in the standard digestion scenario.

## 4. Discussion

Nutritional composition changes were detected in the different processed structures. The main differences relate to chia sprout as compared to the chia seed matrices, particularly as far as moisture is concerned (Table 2). When considering the composition in a dry basis, a reduction in lipid content was observed in the chia sprouts, along with an increase in the protein content. A gradual decrease in lipid content is a well-defined phenomenon occurring in seed sprouting, promoted by the action of endogenous lipases [9,38]. Despite a small part of the resulting free fatty acids being stored in the sprouts, the majority of them are metabolised by the plant by means of one or several pathways [39]. Additionally, the increase in protein after sprouting (compared to seeds and whole flour) reveals a positive balance between protein degradation and protein biosynthesis during germination [39,40]. As regards to carbohydrates, the slightly increased value in partially defatted chia flour is related to the decrease in dry matter as a consequence of oil extraction, while the increase in sprouts could be explained with the formation of new primary cell walls [40]. Many authors found in their studies that germination can gradually accumulate soluble phenolics in germinated edible seeds and sprouts compared with raw seeds. This can be attributed to the de novo synthesis and transformation. Interestingly, an almost twofold increase in antioxidant capacity occurred, suggesting that germination generates new antioxidant compounds other than phenols, including an increase in vitamin content and the activation of the chlorophyll, which has high antioxidant capacity [41]. Finally, germination induces changes in the concentration of minerals, as was observed in calcium concentration of chia sprouts, probably as a consequence of an increase in its extractability [41].

Intact chia seeds not showing lipid or protein digestibility at all supports the inalterability and tightness of the pericarp during the gastric and intestinal digestion of the seeds [6]. It can be therefore stated that intake of integer seeds only provides benefits in terms of mucilage properties, which can be hydrated and gelled. This phenomenon causes an increase in the seeds’ volume, which eventually leads to increased viscosity of the gastric content and satiety, related to gastric wall distention [36,42,43]. Therefore, lipid and protein digestibility in chia seeds requires processing before consumption or incorporation as an ingredient in other food matrices.

A possible explanation for the lipolysis results (Figure 3A) relates to both the optimal physical structure, as mentioned, and the relatively high content of fat (0.325 vs. 0.197 g/g dry matter in partially defatted flour). Previous studies suggested that high lipid concentration in the digestion medium is an enhancing factor for lipolysis [44], and, more specifically, when comparing lipid digestibility of the same food with different fat contents, as in this case [11]. According to this rationale, the fact that partially defatted chia flour reached a lower lipolysis extent than the whole flour (70% and 58% at the normal and altered conditions, respectively) is expected. More recently, Cui et al. [45] also noticed a relationship between lipid content and lipolysis extent. Despite partially defatted flour having a smaller particle size, which could have favoured lipolysis, it seems from this result that the lipid concentration in the digestion medium had a stronger effect on the eventual lipid digestibility. Following this rationale, in chia sprouts, no lipolysis was registered at all for any of the intestinal conditions. As discussed above, sprouts present with a much higher content of water than the seed products, diluting other components, thus resulting in low concentrations of fat in the digestion medium. In addition, when considering the lipid content in dry matter, this fraction is smaller than in the other study counterparts. The previous literature reported that foods with a low content of fat tend to present resistance against lipolysis. Calvo-Lerma et al. [20], in a multi-food study of lipolysis, reported that bread, for example, which had <5% of fat, only showed residual lipolysis extents, while other bakery products with a similar composition but with higher fat content could reach satisfactory levels of lipid digestion. Another explanation for the null lipolysis registered in chia sprouts relates to the possible presence of lipase inhibitory agents, such as phenolic compounds, that could have been developed in the germination process and that proved to have this inhibitory effect [46]. Disregarding chia sprouts, the lowest lipolysis extents (35% in standard and 7% in altered conditions) were reached in chia seeds. As shown in Figure 1, most of the particles were in the range of 1000 µm, which suggests only partial breakdown of the seeds, thus allowing for a smaller fraction with potential accessibility to the enzymes. This result implies that the digestive agents (mechanical agitation, pH, enzymes) were not able to further break down this matrix, at least not to the extent of the other counterparts—whole flour and partially defatted flour—of which the particle sizes were in the range of 300 µm.

Regarding proteolysis throughout the gut, it is a well-defined process; it is characterised by being the main enzymatic phenomenon occurring in the stomach, where pepsin can hydrolyse up to 30%–40% of dietary proteins. Following this, in the small intestine, pancreatic proteases are able to continue with the process, being specific for either entire protein molecules or products of partial hydrolysis such as peptides or amino acids [47]. Two remarkable observations deserve attention regarding gastric lipolysis (Figure 3B). On the one hand, in chia sprouts, complete proteolysis was already achieved at the end of the gastric stage. This result should not be surprising, considering that during grain germination, the storage proteins are hydrolysed into peptides and amino acids by proteolytic enzymes, thus leading to high extents of bioaccessibility of this compound [40,48]. On the other hand, a lower extent of gastric proteolysis was registered in the partially defatted chia flour, probably due to a certain denaturation of proteins during the defatting process. Depending on the type of protein, denaturation has proved to have different effects against digestion. For example, Asensio-Grau et al. [16] found that long exposure to high temperatures in eggs (boiling) leads to lower proteolysis than shorter cooking times (poached). In plant proteins, different species have shown different behaviours towards proteolysis, and, what is more, different digestibility patterns when exposed to pepsin (gastric stage) or trypsin (intestinal stage) [47,49].

Moving onto the effect of intestinal conditions on micronutrients (Table 3), the low calcium bioaccessibility in the germinated chia might be related to the calcium binding by the non-cellulosic fraction of fibre, as previously established [50]. These results, however, must be interpreted with caution: calcium not being detected after the intestinal stage does not necessarily mean that calcium is not available in the in vivo conditions. This would represent a limitation inherent to the commonly used static in vitro digestion models, in which interactions with the products from lipolysis occur because absorption is not simulated, as previously identified by Hu et al. [51]. As referred to the impact of intestinal conditions, more available calcium in the medium was obtained under the altered regime. This result might be explained by the fact that calcium ions have been shown to bind to bile acids, causing precipitation, making the bile concentration lower in altered conditions than in standard conditions [52].

Chia products are of nutritional interest not only because of their content in nutrients, but also for the bioactive compounds with potential antioxidant activity. Any molecule can be considered effective for human health as long as it remains stable after passing through the different stages of digestion [53]. The scientific literature gathers several contributions related to the content of bioactive compounds and antioxidant capacity of chia seeds [3,54]. However, few of them addressed the changes that phenols, the majoritarian antioxidant in chia seeds, display throughout gastrointestinal digestion, and none of them consider altered intestinal conditions (Table 3). Higher polyphenol bioaccessibility being obtained under standard conditions could be expected, given that polyphenols in the matrix are bound to macronutrients (mainly proteins and carbohydrates), so polyphenols release and bioaccessibility are very dependent on macronutrient hydrolysis [55]. The highest extractabilities were detected in the powder forms of chia, whole chia-flour and partially defatted flour. However, in chia seeds, no significant changes in extractability were obtained and, in chia sprouts, the increase was moderated. This result is likely backed up by the fact that unstructured matrices, compared to entire or partially disintegrated structures, allow for the higher release of nutrients to the digestion medium [56]. Focusing on the germination effect, chia sprouts resulted in more bioaccessible polyphenols than chia seeds. This finding is in accordance with the evidence that nutrients in sprouted products are more bioaccessible and bioavailable than the seeds they come from Pająk et al. [22], but the milling process seems to be the most determinant in this regard.

A possible explanation for the overall decrease in antioxidant activity points to other bioactive components with antioxidant capacity being destroyed during in vitro digestion, despite polyphenols being maintained (Table 3). The reduction in the antioxidant capacity could also also related to the released bioactive molecules interacting with each other in an antagonistic sense. A previous study compared the antioxidant activity of different polyphenols and their combinations [57]. The results show that the majority of DPPH scavenging activities in these combinations promoted antagonistic effects in most of the cases, leading to reduced total antioxidant capacity when different phenolic compounds are co-digested [57]. Overall, the decrease in antioxidant activity was more noticeable in the altered intestinal conditions. This finding is backed up by the fact that in the altered digestion conditions, less matrix degradation occurred, thus less release of compounds to the digestion medium was achieved. Of note, only the liquid phase of the digesta was considered for the determination of antioxidant activity, excluding the solid phase, where part of the non-bioaccessible bioactive compounds could have remained. In contrast, another study assessed both the solid and liquid phases in chia seeds after intestinal digestion, registering an increase in total antioxidant activity [7]. Nonetheless, a closer approach to the in vivo situation includes the assessment of the liquid phase only, where the bioaccessible fraction of digestion products remains.

## 5. Conclusions

The premise for conducting the present study was the high potential of chia seeds in providing nutrients with beneficial effects on health. As discussed, these nutrients in whole intact seeds can barely be accessed throughout digestion and thus the consumption of whole seeds, as such, should not be recommended. High contents of fibre would be guaranteed, however. In contrast, different processing techniques led to variable effects on nutrient digestibility and bioaccessibility. According to the results of the present study, germination promotes the hydrolysis of chia proteins, making them completely digestible, and causes a significant increase in polyphenols and calcium concentration in chia sprouts. In the case of chia seed products, the particle size and treatments such as defatting or milling had a significant impact on the digestibility of macronutrients and extractability of calcium and polyphenols. The in vitro digestion study of the different products evidences the degradation of polyphenols and then the decrease in antioxidant activity in spite of the increase in extractability. On balance, then, the results from the present study allow us to know in detail the impact of milling and sprouting in order to make knowledge-based recommendations about the nutritional benefits of the different products. Nevertheless, the relevance in nutrition regarding all these findings should be compared with in vivo studies in a further step.

## Figures and Tables

**Table 1:** Overview of the parameters defining the oral, gastric and intestinal stages of the in vitro digestion experiments.

Stage	Oral	Gastric	Intestinal
Input	Food sample (5 g)	Oral bolus (10 mL)	Gastric chyme (20 mL)
Digestive fluid	Simulated salivary fluid (5 mL)	Simulated gastric fluid (10 mL)	Simulated intestinal fluid (20 mL)
Composition of digestive fluid	α-amylase (75 U/mL)	Pepsin (2000 U/mL)	Pancreatin (Lipase: 30 LU/mL; Protease 1.8 U/mL; α-amylase: 12 U/mL) Bile salts (1 or 10 mM *)
Medium pH	7	3	6 or 7 *
Temperature	37 °C	37 °C	37 °C
Duration	5 min	120 min	120 min
Mechanical force	Grinder pulses	Head-over heels agitation (100 rpm)	Head-over heels agitation (60 rpm)

* depending on the experimental design: pH 7—bile salts 10 mM in case of simulating normal conditions; pH 6—bile salts 1 mM in case of altered conditions.

**Table 2:** Nutritional composition of chia seeds and whole flour, partially defatted chia flour and sprouts (data are expressed as mean (SD) from three repetitions).

	Chia Seeds and Whole Flour	Partially Defatted Flour	Chia Sprouts
Moisture (g/g dry matter)	0.065 (0.008) ^a^	0.068 (0.009) ^a^	9.87 (0.010) ^b^
Lipids (g/g dry matter)	0.325 (0.011) ^c^	0.197 (0.007) ^b^	0.097 (0.003) ^a^
Protein (g/g dry matter)	0.201 (0.007) ^a^	0.291 (0.011) ^c^	0.229 (0.009) ^b^
Carbohydrate (g/g dry matter)	0.472 (0.016) ^a^	0.501 (0.017) ^b^	0.644 (0.022) ^c^
Calcium (mg/g dry matter)	6.46 (0.046) ^b^	3.45 (0.032) ^a^	7.26 (0.18) ^c^
Polyphenols (mg GA eq./g dry matter)	1.78 (0.03) ^b^	1.24 (0.02) ^a^	2.87 (0.06) ^c^
Antioxidant activity (mg TX eq./g dry matter)	3.49 (0.11) ^b^	2.58 (0.09) ^a^	5.69 (1.6) ^c^

Values sharing a same letter in the same row are not significantly different (*p* < 0.05). GA eq., gallic acid equivalent; TX, trolox equivalent.

**Table 3:** Concentration of calcium, polyphenols and antioxidant activity in the bioaccessible fraction after in vitro digestion under standard and altered intestinal conditions (data are expressed as mean values (SD) from three repetitions).

	Intestinal Conditions	Chia Seeds	Whole Chia Flour	Partially Defatted Chia Flour	Chia Sprouts
Calcium (mg/g dry matter)	Standard	3.82 (0.03) ^Ca^	4.22 (0.03) ^Da^	2.09 (0.07) ^Ba^	0.15 (0.02) ^Aa^
	Altered	3.77 (0.04) ^Ca^	4.28 (0.02) ^Da^	2.07 (0.03) ^Ba^	0.32 (0.04) ^Ab^
Polyphenols mg (GA eq./g dry matter)	Standard	1.81 (0.17) ^Ab^	6.13 (0.38) ^Db^	2.67 (0.28) ^Bb^	4.41 (0.51) ^Cb^
	Altered	1.51 (0.15) ^Aa^	3.91 (0.16) ^Da^	2.13 (0.13) ^Ba^	3.55 (0.13) ^Ca^
Antioxidant activity (mg TX eq./g dry matter)	Standard	1.17 (0.06) ^Ab^	4.8 (0.38) ^Cb^	3.97 (0.75) ^Bb^	4.21 (1.17) ^Bb^
	Altered	1.03 (0.09) ^Aa^	3.31 (0.33) ^Ca^	2.53 (0.39) ^Ba^	2.23 (0.74) ^Ba^

Different capital letters (A–D) mean significant differences (*p* < 0.05) between chia products (chia seeds, whole chia flour, partially defatted chia flour and chia spouts). Different lowercase letters (a, b) mean significant differences (*p* < 0.05) between intestinal conditions (standard and alternated). GA eq., gallic acid equivalent; TX eq., trolox equivalent.
